# Single Port Robotic Nephrectomy via lower anterior retroperitoneal approach: feasible, safe and effective option in surgically complex patients

**DOI:** 10.1590/S1677-5538.IBJU.2024.0420

**Published:** 2024-08-15

**Authors:** Angelo Orsini, Francesco Lasorsa, Gabriele Bignante, Jamie Yoon, Kyle Anna Dymanus, Edward E. Cherullo, Riccardo Autorino

**Affiliations:** 1 Rush University Department of Urology Chicago IL USA Department of Urology, Rush University, Chicago, IL, USA; 2 D’Annunzio University of Chieti-Pescara Department of Urology Chieti Italy Department of Urology, D’Annunzio University of Chieti-Pescara, Chieti, Italy; 3 University of Bari "Aldo Moro" Andrology and Kidney Transplantation Unit Department of Precision and Regenerative Medicine and Ionian Area-Urology Bari Italy Division of Urology, Department of Precision and Regenerative Medicine and Ionian Area-Urology, Andrology and Kidney Transplantation Unit, University of Bari "Aldo Moro", Bari, Italy; 4 University of Turin Department of Oncology Division of Urology Turin Italy Division of Urology, Department of Oncology, University of Turin, San Luigi Gonzaga Hospital, Turin, Italy

## Abstract

**Purpose:**

Minimally invasive radical nephrectomy is often preferred for larger renal tumours not suitable for partial nephrectomy (1). When performed with a multiport robot, the procedure is routinely performed with a transperitoneal approach, with recent studies highlighting important factors for surgical outcomes, including predictive factors (2), segmental artery unclamping techniques (3), and comparisons of robotic techniques (4).

This video shows that SP Robot-Assisted Radical Nephrectomy (RARN) via a lower anterior approach is valuable in challenging cases.

**Materials and Methods:**

We performed SP-RARN on two complex patients using a retroperitoneal lower anterior approach. The first patient, a 54-year-old female with a BMI of 36.8 kg/m^2^, had a ventral hernia and bowel obstruction history, with a 9 cm right middle kidney mass. The second patient, a 58-year-old male with a BMI of 31.19 kg/m^2^, had ESRD and was on peritoneal dialysis for 8 years, with a 3.4x3.7 cm mass in the right superior pole, suspected to be RCC. The surgical technique is detailed in the video.

**Results:**

Both procedures were successful, with operative times of 173 and 203 minutes and blood loss of 150 mL. No complications occurred. Patients were discharged after 31 and 38 hours, respectively. Histopathology confirmed RCC. At the 3-month follow-up, no complications or readmissions were reported. Second patient continued peritoneal dialysis without issues.

**Conclusion:**

Retroperitoneal SP-RARN via the lower anterior approach avoids the peritoneal cavity, making it suitable for certain patients. In these patients, more so than in others, this procedure is feasible, safe, and less morbid than the standard multiport approach.

**Available at:** http://www.intbrazjurol.com.br/video-section/20240420_Autorino_et_al
